# Mass spectrometry data of *in vitro* and *in vivo* pig digestion of skim milk powder

**DOI:** 10.1016/j.dib.2018.09.089

**Published:** 2018-10-16

**Authors:** Lotti Egger, Patrick Schlegel, Christian Baumann, Helena Stoffers, Dominik Guggisberg, Cédric Brügger, Desirée Dürr, Peter Stoll, Guy Vergères, Reto Portmann

**Affiliations:** a Agroscope, Schwarzenburgstr. 161, Bern 3003, Switzerland; b Agroscope, Tioleyre 4, 1725 Posieux, Switzerland

## Abstract

The data in this article are related to the research article entitled “Physiological comparability of the harmonized INFOGEST *in vitro* digestion method to *in vivo* pig digestion” (Egger et al., 2012). In this article, proteins identified in the different sections of pig skim milk powder (SMP) digestion are presented. In addition to the exemplary β-casein profiles of the paper, the peptide patterns of the other most abundant milk proteins during *in vivo* digestion in individual pigs are shown as heatmaps and line graphs. These data clearly reveal the digestion resistant protein regions and illustrate the variability between the pigs in the different sampling sections. Moreover, peptide patterns of the same SMP proteins comparing the harmonized *in vitro* digestion (IVD) with pig *in vivo* digestion show the physiological relevance of the IVD protocol. Finally, correlation coefficients were calculated to indicate similarities between pig sampling sections and gastric and intestinal IVD endpoints.

**Specifications table**

**Table:** 

Subject area	Biology
More specific subject area	Proteomics and biochemistry
Type of data	Table, figures
How data was acquired	High pressure liquid chromatography coupled to a mass spectrometer using an electron spray ionization interface (LTQ, Thermo Scientific)
Data format	analyzed
Experimental factors	Digested samples were filtered through cut-off filters with a pore size of 30 kDa and directly injected to the mass spectrometer
Experimental features	MS/MS raw files were merged with Mascot Daemon, and identification search was performed with Mascot.
Data source location	
Data accessibility	Data is with this article

**Value of the data**

• The amino acid count method allows a semi-quantitative assessment of peptides after *in vitro* and *in vivo* digestion.
• Illustration of the variability between samples and between different experimental protocols.
• Peptide patterns allow the visualization of digestion resistant regions within dairy proteins.

## 1 Data

**Fig. 1:**
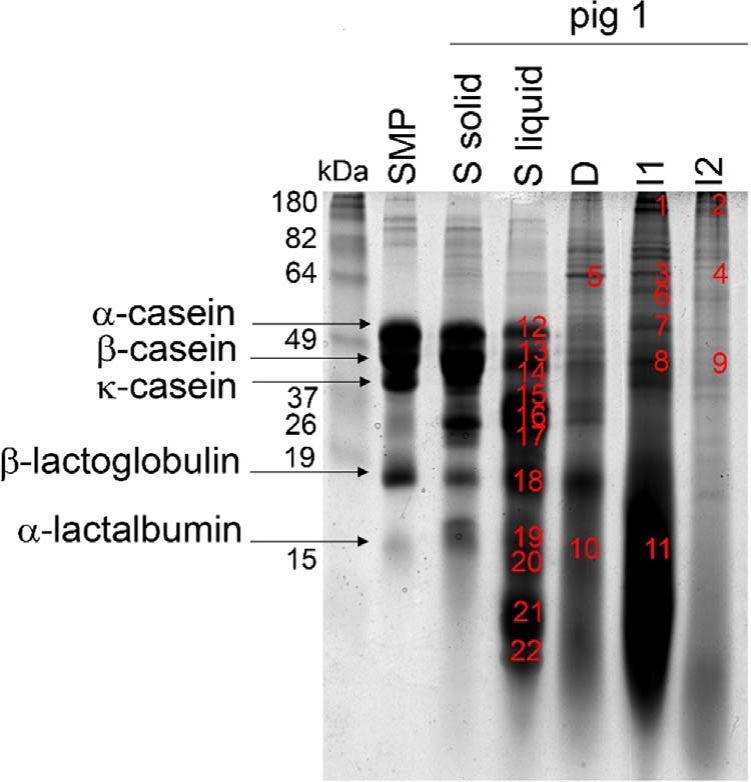
Protein identifications after SMP digestion in individual pigs. The indicated spots were identified with mass spectrometry (MS). Spots 1–11 are digestive enzymes or proteins from the pigs, and spots 12–22 are milk proteins listed in Table 1. Band labeling: Skim milk powder (SMP), stomach solid (S solid), duodenum (D, 0–30 cm after the stomach), proximal jejunum (I1, 50–150 cm of the small intestine), median jejunum (I2, 200–300 cm of the small intestine), late jejunum (I3, the last part of the small intestine) and ileum (I4).

The SDS gel in Fig. 1 shows the protein bands from undigested SMP and its hydrolysis during *in vivo* pig digestion. The different bands were identified with mass spectrometry (MS) as previously described [2]. Spots 1–11 are digestive enzymes or proteins originating from the pigs, and spots 12–22 are milk proteins listed in Table 1.

**Table 1:** Protein identifications after SMP digestion in individual pigs. The band number corresponds to the numbers in Fig. 1. M: major (>30% of total peptide intensity) identified protein in the corresponding band; X: minor identified protein in the same band.

**Fig. 2:**
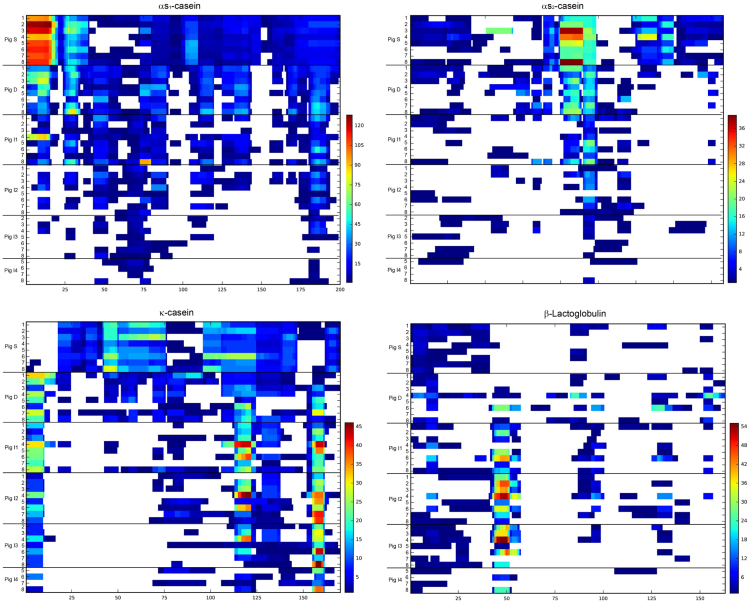
Shows the peptide generation and variability of the different sampling sections of *in vivo* pig digestion. Peptide patterns from all samples for αs_1_-casein, αs_2_-casein, κ-casein and β-lactoglobulin. The sections shown are from the stomach (S), duodenum (D), jejunum (I1–3) and ileum (I4). The frequencies of the peptides are visualized using the indicated color code. White stretches indicate that no peptides were identified for the corresponding sequences. The protein sequence is on the *x*-axis, and the different animals separated by sampling sections are on the *y*-axis.

**Fig. 3:**
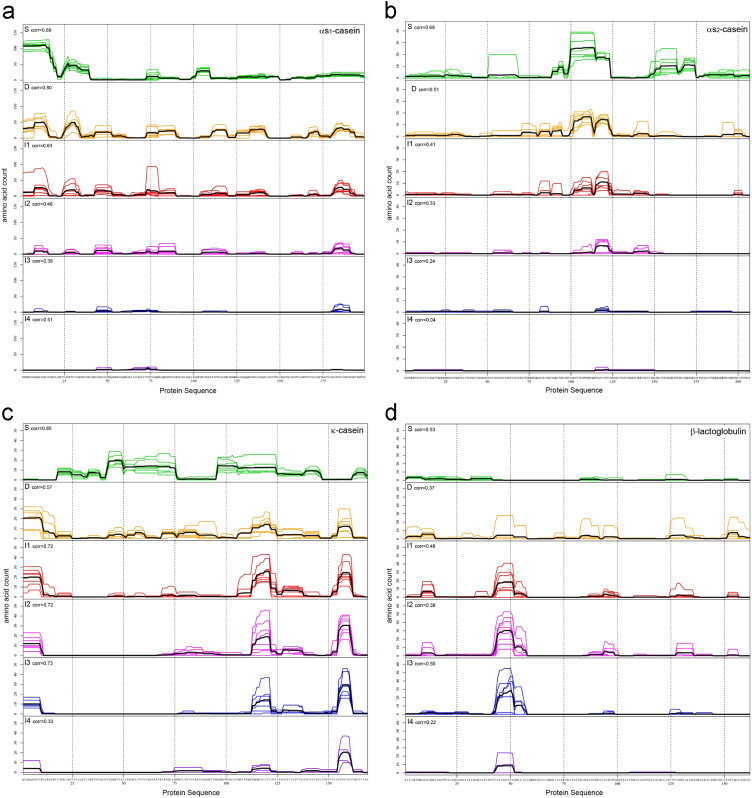
Peptide generation and variability of the different sampling sections of *in vivo* pig digestion. The average peptide pattern generated for αs_1_-casein (a), αs_2_-casein (b), κ-casein (c), and β-lactoglobulin (d) included all pig samples. The correlation coefficient between the different animals is indicated per sampling segment. The protein sequence is shown on the *x*-axis, and the frequency of an identified amino acid within the protein is shown on the *y*-axis.

The peptide generation during SMP digestion and the variability between individual pigs in the different sampling sections was shown as heatmaps (Fig. 2) and line graphs (Fig. 3). These graphs also highlight the digestion resistant regions of the different SMP proteins (αs_1_-casein, αs_2_-casein, κ-casein and β-lactoglobulin). The digestion patterns were obtained with the amino acid count method.

**Fig. 4:**
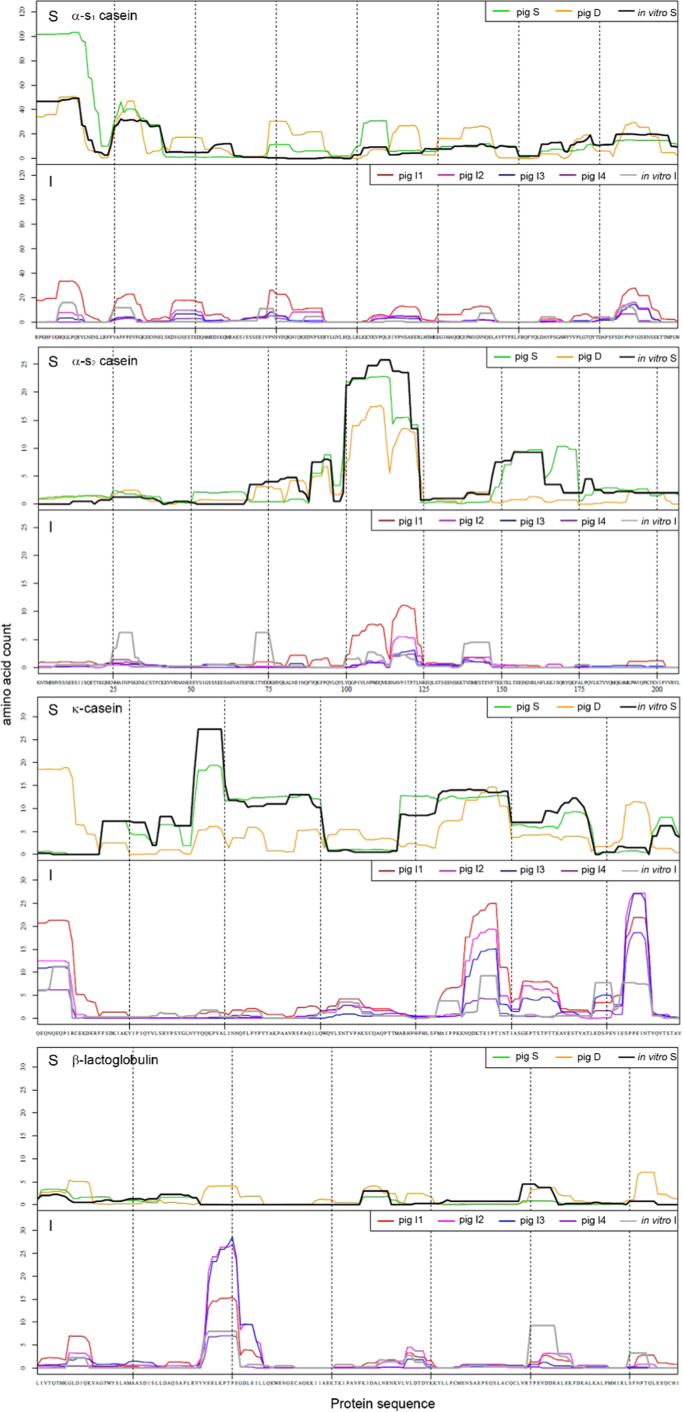
Comparison of peptide patterns between *in vivo* and *in vitro* digestion. The mean peptide patterns of αs_1_-casein, αs_2_-casein, κ-casein and β-lactoglobulin from IVD were compared with the *in vivo* gastric (upper graph, pig S, pig D, *in vitro* S) and the intestinal phases. The protein sequence is shown on the *x*-axis, and the frequency of an identified amino acid within the protein is shown on the *y*-axis.

The average patterns of the different pig sampling sections were compared to the endpoints of gastric and intestinal digestion of the same SMP, using the harmonized IVD protocol (Fig. 4). The endpoints of the gastric digestion were directly compared to the stomach and the duodenal samples of the pig trial, the intestinal digestion was matched to the pig intestinal sections spanning from jejunum to ileum.

**Fig. 5:**
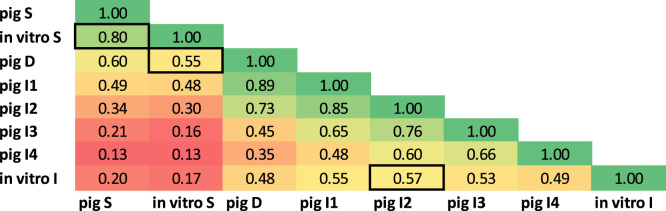
Correlation between *in vivo* and *in vitro* digestion. Correlations were calculated comprising all pig sampling sections and IVD gastric and intestinal samples. Best correlations of *in vitro* gastric or intestinal *versus* the corresponding *in vivo* samples are highlighted with a black frame.

Correlations were calculated between all the protein patterns revealing best matches among the *in vivo* and *in vitro* data (Fig. 5). This correlation included all mayor SMP proteins.

## 2 Experimental design, materials and methods

### 2.1 Amino acid count method

Digested samples were passed through cut-off filters (30 kDa), and subsequently separated by high pressure liquid chromatography (solvent gradient H_2_O (A) to acetonitrile (B), both with 0.1% formic acid, 0–15 min: 5–60% (B), 15–20 min: 60–95% (B)), coupled to a mass spectrometer using an electron spray ionization interface [2]. The samples were measured in four overlapping narrow-mass windows for peptide fragmentation over a total range of 290–1300 m/z (*i.e.* 290–410, 390–610, 590–910, and 890–1300). The minimal signal intensity was set to 500 for MS/MS spectra generation. The obtained raw files were merged with Mascot Daemon, prior to the identification search with Mascot, using a milk protein database from different species. Peptides with a minimal length of 5 amino acids and an ion score cut-off of 20 were considered. Identified peptides were aligned to the protein sequence. Peptides are typically identified multiple times per MS/MS run, therefore a relative quantification was introduced by summing up the number of times each amino acid was identified within a milk protein, defined as amino acid count. Fig. 2 shows the heatmap representation, using a color code from low abundance (blue), to medium abundance (green), and high abundance (red). White stretches indicate non-identified sequences.

All other methods are described in the original research article [1].
