# Supplementary material for: Mass spectrometry data of in vitro and in vivo pig digestion of skim milk powder
Source: Data Brief. 2018 Oct 16;21:911–7. doi: 10.1016/j.dib.2018.09.089 (PMC6222262; doi:10.1016/j.dib.2018.09.089)
Supplement: Supplementary file 1 — Supplementary material [file mmc1.docx]

**P.P. Agroscope, egch, Schwarzenburgstrasse 161, 3003 Bern / Switzerland**

Editor‐in-Chief, Food Research International

Bern, 14. February 2017

Reference:

Your reference:

Our reference: egch

Contact person:

Liebefeld, 14. February

Dear Professor Sant’Ana,

We would like to submit the revised DiB article *"Mass spectrometry data of in vitro and in vivo pig digestion of skim milk powder".* We apologize for the delay of this resubmission and hope that you still can reconsider it.

We have changed the references according to the example provided in the e-mail.

We hope that you find that the DiB article is now suited for publication in *Food Research International*. As requested, we confirm that the manuscript has not been previously published in any language anywhere and is not under simultaneous consideration by another journal. The corresponding author and all of the other authors have read and approved the final submitted manuscript. Moreover, there is no conflict of interest by any of the authors of the paper.

Sincerely,

Charlotte Egger
